# Klotho alleviates indoxyl sulfate-induced heart failure and kidney damage by promoting M2 macrophage polarization

**DOI:** 10.18632/aging.103183

**Published:** 2020-05-28

**Authors:** Jing Lv, Jin Chen, Minjia Wang, Fei Yan

**Affiliations:** 1 Department of General Practice, Zhejiang Hospital, Hangzhou 310013, Zhejiang, P.R. China; 2 Department of Critical Care Medicine, Zhejiang Hospital, Hangzhou 310013, Zhejiang, P.R. China

**Keywords:** chronic kidney disease, cardiovascular disease, Klotho, indoxyl sulfate, macrophage polarization

## Abstract

Indoxyl sulfate (IS) is a protein-bound uremic toxin that can accumulate in patients with chronic kidney disease (CKD) or acute kidney injury (AKI) and cause kidney and cardiac dysfunction. Klotho is an anti-aging protein that has reno- and cardio-protective effects. We investigated whether Klotho could alleviate IS-induced heart failure and kidney damage by regulating macrophages, which play a key role in the inflammatory response in CKD and AKI. Treatment of THP-1-derived macrophages with IS induced the production of the pro-inflammatory cytokines TNFα, IL-6, and IL-1β, and stimulated M1 polarization. Additionally, IS induced downregulation of Klotho expression in macrophages. Overexpression of Klotho suppressed the IS-induced inflammatory response in macrophages by stimulating M2 polarization. It also alleviated IS-induced cardiac hypertrophy and renal fibrosis in mice. A reduction in IS-induced phosphorylation of NF-kB p65 was observed in response to Klotho overexpression, suggesting that Klotho alleviates kidney and cardiac injury by inactivating NF-kB signaling and promoting macrophage M2 polarization.

## INTRODUCTION

Chronic kidney disease (CKD) and acute kidney injury (AKI) have a high global prevalence and economic burden [1]. CKD is defined as a decreased estimated glomerular filtration rate (GFR, <60 mL/min/1.73 m^2^) for at least 3 months and a gradual and permanent loss of kidney function over the course of months or years [2, 3]. AKI is characterized by an abrupt decline in renal function [4]. Cardiovascular disease (CVD) is a serious complication in patients with CKD and AKI, and is the main cause of death among patients on dialysis [5–7]. Reduced GFR has been correlated with an increased risk of CVD among CKD patients [8, 9].

Macrophages are the main contributors to the inflammatory response in CKD and AKI [10–12]. They play a dual role in AKI through polarization towards the pro-inflammatory M1 (classically activated) and anti-inflammatory M2 (alternatively activated) phenotypes [4, 13, 14]. Previous studies have demonstrated that M2 macrophage polarization is important for cardiac repair and recovery from kidney injury in AKI patients [15, 16].

Indoxyl sulfate (IS) is a protein-bound uremic toxin present at higher levels in CKD patients [17]. Reduced excretion of IS has been observed in CKD patients as a consequence of a reduced GFR, and the resulting accumulation of IS can affect both cardiac and kidney function [18–20]. IS was found to accelerate the development of renal fibrosis in CKD patients [19]. It also promoted cardiac hypertrophy and inflammation [19].

Klotho is an anti-aging protein that is predominantly expressed in renal tubular epithelial cells where it has been shown to have reno-protective effects [21, 22]. Reduced expression of Klotho has been observed in CKD patients [23]. Restoration of Klotho expression could prevent cell senescence and alleviate vascular calcification in CKD [24, 25]. In addition, jia et al found that decreased klotho expression accelerated the progression of diabetic kidney disease via promoting M1 polarization [26]. However, the molecular mechanisms by which Klotho protects the injury of kidney and cardiac in CKD remains unclear. In this study, we investigated whether Klotho exerts reno- and cardio-protective effects by stimulating macrophage polarization towards the anti-inflammatory M2 phenotype.

## RESULTS

### IS stimulates pro-inflammatory cytokine production and promotes M1 macrophage polarization

**Figure 1:**
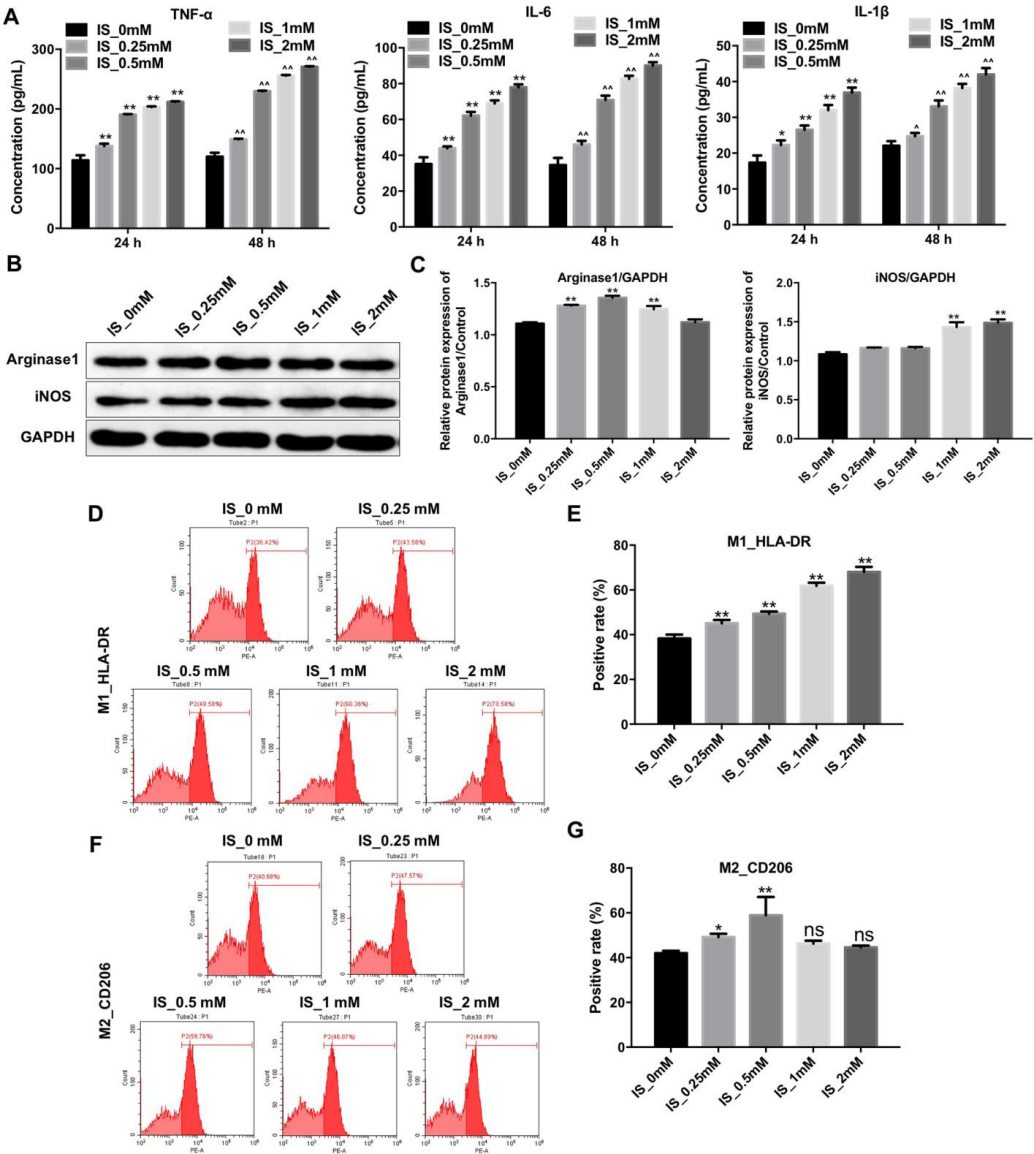
**IS stimulates the production of pro-inflammatory cytokines and promotes M1 macrophage polarization.** (**A**) THP-1 cells were exposed to PMA (160 nM) for 48 h and then incubated in PMA-free medium for 24 h followed by the indicated concentrations of IS (0, 0.25, 0.5, 1, or 2 mM) for 24 and 48 h. The levels of TNFα, IL-6, and IL-1β were then analyzed by ELISA. (**B**) THP-1 cells were exposed to PMA (160 nM) for 48 h and then incubated in PMA-free medium for 24 h followed by the indicated concentrations of IS (0, 0.25, 0.5, 1, or 2 mM) for 24 h. Arginase1 and iNOS expression was analyzed by western blotting. (**C**) Arginase1 and iNOS expression was normalized to GAPDH. (**D**, **E**) Representative FACS plots for HLA-DR, a marker of M1 macrophages. Percentages of HLA-DR+ cells detected by FACS. (**F**, **G**) Representative FACS plots for CD206, a marker of M2 macrophages. Percentages of CD206+ cells detected by FACS. **P < 0.01 vs. IS-0 mM group.

IS can stimulate the release of pro-inflammatory factors [19]. We found that IS stimulated the production of several pro-inflammatory cytokines (TNFα, IL-6, and IL-1β) in THP-1-derived macrophages (Figure 1A). The expression of iNOS (M1 biomarker) increased after treatment of the cells with 2 mM IS. However, it had no effect on Arginase1 expression (M2 biomarker) (Figure 1B, 1C).

We performed fluorescence activated cell sorting (FACS) to investigate the effects of IS on macrophage polarization. We found that treatment of THP-1-derived macrophages with 2 mM IS resulted in an increase in the proportion of HLA-DR^+^ cells (M1 macrophages) (Figure 1D, 1E). However, the proportion of CD206^+^ cells (M2 macrophages) was comparable between cells treated with 2 mM vs. 0 mM IS (Figure 1F, 1G). These data indicated that IS could promote M1 macrophage polarization.

### IS reduces Klotho expression in THP-1-derived macrophages

**Figure 2:**
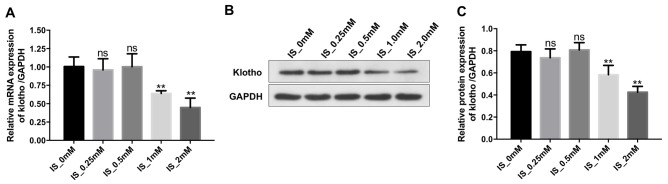
**IS decreases the expression of Klotho in macrophages.** THP-1 cells were exposed to PMA (160 nM) for 48 h and then incubated in PMA-free medium for 24 h followed by the indicated concentrations of IS (0, 0.25, 0.5, 1, or 2 mM) for 24 h. (**A**) RT-qPCR analysis of Klotho expression in cells. (**B**) Western blot analysis of Klotho protein expression. (**C**) Klotho expression was normalized to GAPDH. **P < 0.01 vs. IS-0 mM group.

We next performed quantitative reverse transcription PCR (RT-qPCR) and western blotting to evaluate the relationship between IS and Klotho expression in macrophages. Treatment of THP-1-derived macrophages with 2 mM IS resulted in a decrease in Klotho expression (Figure 2A–2C). Therefore, 2 mM IS was utilized for all subsequent experiments.

### Overexpression of Klotho suppresses the IS-induced inflammatory response by promoting M2 macrophage polarization

**Figure 3:**
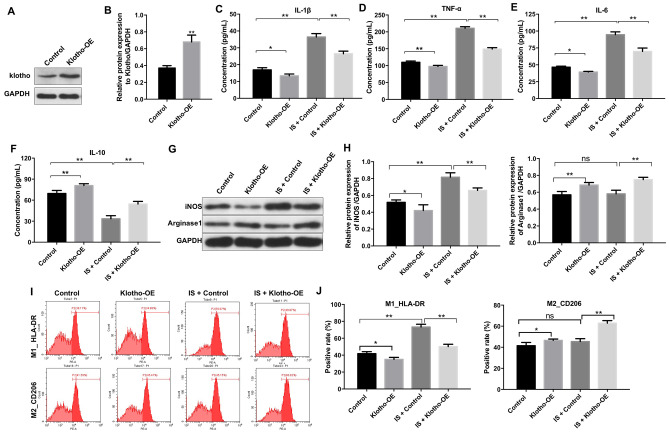
**Overexpression of Klotho suppresses the IS-induced inflammatory response in macrophages by stimulating M2 polarization.** (**A**) THP-1 cells were exposed to PMA (160 nM) for 48 h, incubated in PMA-free medium for 24 h, and then transfected with the Klotho expression plasmid for 24 h. Klotho expression in cells was evaluated by western blotting. (**B**) Klotho expression was normalized to GAPDH. (**C**–**F**) THP-1 cells were exposed to PMA (160 nM) for 48 h, incubated in PMA-free medium for 24 h. Subsequently, cells were transfected with Klotho expression plasmid or treated with 2 mM IS for 24 h respectively. Meanwhile, cells were transfected with Klotho expression plasmid for 24 h in the presence of 2 mM IS. The levels of IL-10, IL-6, TNFα, and IL-1β in cells were evaluated by ELISA. (**G**) The expression of iNOS and Arginase1 in cells was analyzed by western blotting. (**H**) The expression of iNOS and Arginase1 in cells was normalized to GAPDH. (**I**) Representative FACS plots for HLA-DR, a marker of M1 macrophages, and CD206, a marker of M2 macrophages. (**J**) The percentages of HLA-DR- and CD206+ cells were detected by FACS. *P < 0.05 and **P < 0.01.

We investigated the effects of Klotho overexpression on IS-stimulated macrophages. An increase in Klotho expression was observed in macrophages following transfection with the Klotho construct for 24 h as expected (Figure 3A, 3B). Treatment with IS resulted in a reduction in the level of the anti-inflammatory factor IL-10, and an increase in the levels of the pro-inflammatory factors IL-1β, IL-6, and TNFα in macrophages. These effects were alleviated by overexpression of Klotho, suggesting that overexpression suppresses the IS-induced inflammatory response by promoting M2 polarization (Figure 3C–3F).

Overexpression of Klotho reversed the IS-induced increase in iNOS expression (Figure 3G, 3H). It also resulted in an increase in Arginase1 expression in IS-stimulated macrophages (Klotho + IS group) compared to macrophages treated with IS alone (IS group) (Figure 3G, 3H). Interestingly, Klotho overexpression resulted in a decrease in the proportion of HLA-DR+ cells (M1 macrophages) (Figure 3I, 3J) and an increase in the proportion of CD206+ cells (M2 macrophages) in the Klotho + IS compared to IS group (Figure 3I, 3J). These data suggested that overexpression of Klotho suppressed M1 and promoted M2 macrophage polarization.

### Overexpression of Klotho suppresses the IS-induced inflammatory response *in vivo* by promoting M2 macrophage polarization

**Figure 4:**
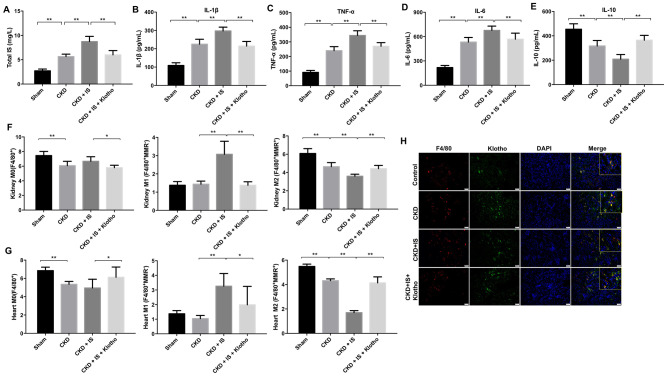
**Overexpression of Klotho suppresses the IS-induced inflammatory response *in vivo* by promoting M2 macrophage polarization.** (**A**) The total IS concentration (mg/L) was measured by UPLC. (**B**–**E**) The levels of IL-1β, TNFα, IL-6, and IL-10 in serum from mice were analyzed by ELISA. (**F**) Representative FACS plots for M0 (F4/80^+^), M1 (F4/80^+^MMR^-^), and M2 (F4/80^+^MMR^+^) macrophages. The percentages of F4/80^+^, F4/80^+^MMR^-^, and F4/80^+^MMR^+^ cells were evaluated in kidney tissue using FACS. (**G**) The percentages of F4/80^+^, F4/80^+^MMR^-^, and F4/80^+^MMR^+^ cells were evaluated in heart tissue using FACS. (**H**) Quantification of relative expression based on F4/80, Klotho, and DAPI staining. *P < 0.05, **P < 0.01.

We established an IS-induced mouse model of heart failure and kidney damage to investigate the role of Klotho *in vivo*. The levels of IS in serum were higher in mice with CKD compared to controls (Figure 4A). Overexpression of Klotho resulted in a decrease in serum IS levels in the CKD + IS + Klotho compared to CKD + IS treatment group (Figure 4A). Additionally, Klotho overexpression resulted in a decrease in serum IL-1β, TNFα, and IL-6, and an increase in serum IL-10 in the CKD + IS + Klotho compared to CKD + IS group (Figure 4B–4E). Thus, overexpression of Klotho suppressed the IS-induced inflammatory response *in* *vivo*.

We investigated the effects of Klotho overexpression on macrophage polarization *in vivo*. Klotho overexpression suppressed M1 and promoted M2 polarization in kidney and heart tissue from mice in the CKD + IS + Klotho compared to CKD + IS group (Figure 4F, 4G). Moreover, the IS-induced reduction of Klotho protein expression in macrophages was reversed following transfection with the Klotho expression plasmid (Figure 4H). These data indicated that Klotho overexpression stimulated M2 macrophage polarization in a mouse model of IS-induced heart failure and kidney damage.

### Overexpression of Klotho alleviates IS-induced heart failure and kidney damage *in vivo*

**Figure 5:**
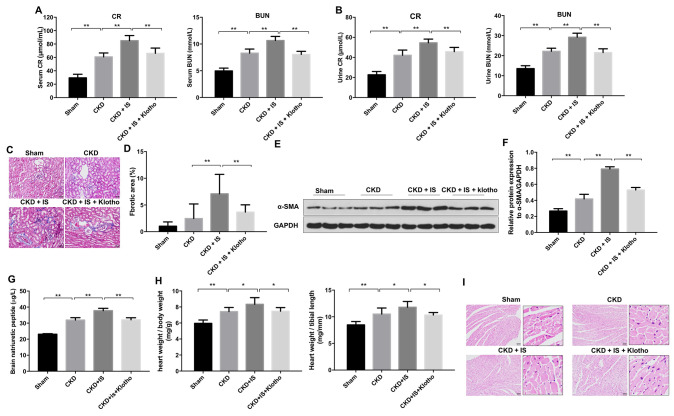
**Overexpression of Klotho alleviates IS-induced heart failure and kidney damage *in vivo*.** (**A**) Serum CR and BUN levels detected by ELISA. (**B**) Urine CR and BUN levels detected by ELISA. (**C**, **D**) Analysis of renal fibrosis by Masson staining. (**E**) Expression of α-SMA in kidney tissue evaluated by western blotting. (**F**) Expression of α-SMA relative to GAPDH in kidney tissue. (**G**) Serum BNP level detected by ELISA. (**H**) Heart weight was calculated in each treatment group. (**I**) Heart tissue was stained with hematoxylin and eosin. *P < 0.05, **P < 0.01.

Overexpression of Klotho resulted in a decrease in urine and serum blood urea nitrogen (BUN) and creatinine (CR) levels in mice in the CKD + IS + Klotho compared to CKD + IS group (Figure 5A, 5B). An increase in the rate of fibrosis in mouse kidney tissue was observed in the CKD + IS compared to CKD group, which was alleviated following infection with Ad-Klotho (Figure 5C, 5D). Finally, Klotho overexpression resulted in downregulation of α-SMA expression in kidney tissue in the CKD + IS + Klotho compared to CKD + IS group (Figure 5E, 5F). These data demonstrated that Klotho overexpression inhibited IS-induced renal fibrosis *in vivo*.

Overexpression of Klotho resulted in a decrease in BNP level in mice in the CKD + IS + Klotho compared to CKD + IS group (Figure 5G). In addition, we observed higher relative heart weights in the CKD + IS compared to CKD group. A reduction in heart weight was observed in mice infected with Ad-Klotho (Figure 5H). We also observed an increase in cardiomyocyte size in the CKD + IS compared to CKD group. Overexpression of Klotho resulted in a decreased in cardiomyocyte size in the CKD + IS + Klotho compared to CKD + IS group (Figure 5I). Thus, Klotho overexpression inhibited IS-induced cardiomyocyte hypertrophy *in vivo*.

### Overexpression of Klotho alleviates heart failure and kidney damage *in vitro* and *in vivo* through inactivating the NF-kB pathway

**Figure 6:**
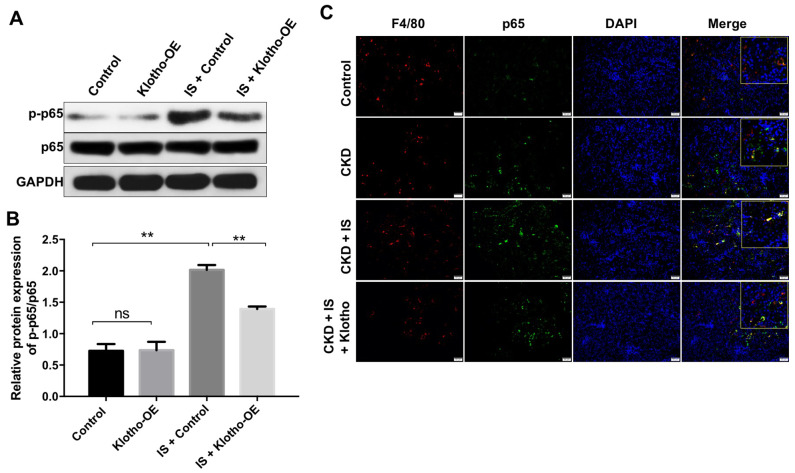
**Overexpression of Klotho alleviates IS-induced heart failure and kidney damage *in vitro* and *in vivo* by activating the NF-kB pathway.** (**A**) THP-1 cells were exposed to PMA (160 nM) for 48 h, incubated in PMA-free medium for 24 h, and then transfected with the Klotho expression plasmid for 24 h. The expression of p-p65 in cells was evaluated by western blotting. (**B**) The relative expression of p-p65 was evaluated normalized to p65. (**C**) Quantification of relative expression based on F4/80, p65 and DAPI staining in mouse kidney tissue. **P < 0.01.

We next investigated the mechanism by which Klotho overexpression revered IS-induced heart failure and kidney damage. Klotho overexpression resulted in downregulation of IS-induced phosphorylation of NF-kB p65 *in vitro* and *in vivo* (Figure 6A–6C). These data suggested that overexpression of Klotho alleviates heart failure and kidney damage by inactivating the NF-kB pathway.

## DISCUSSION

Evidence has been shown that Klotho could protect against IS-induced cardiac injury in mice with CKD [27]. Meanwhile, it has been shown that downregulation of klotho could accelerate the progression of diabetic kidney disease via promoting M1 polarization [26]. Therefore, in the present study, we aimed to explore whether Klotho exhibited the reno-protective and cardioprotective roles via regulating macrophage polarization. We found that Klotho overexpression can suppress the IS-induced inflammatory response by promoting M2 macrophage polarization. Additionally, it reduces IS-induced renal fibrosis and cardiac hypertrophy in a mouse model of heart failure and kidney damage. Reduced renal function in CKD (i.e. a reduced GFR) can lead to the accumulation of IS, which can contribute to the development of CVD [28]. IS was previously found to induce renal fibrosis and cardiac hypertrophy in CKD [29, 30]. IS increased the expression of MCP-1 and α-SMA, markers of inflammation and fibrosis, respectively, in renal proximal tubular cells [31]. Consistent with these previous studies, we demonstrated that IS induces renal fibrosis and cardiomyocyte hypertrophy *in vivo*. IS was found to promote inflammation through activation of the NF-κB signaling pathway in cardiomyocytes [32]. Here, we demonstrated that IS increased the levels of NF-kB *in vitro* and *in vivo*. Therefore, IS could induce heart failure and kidney damage by activating the NF-kB signaling pathway.

Klotho was shown to protect against IS-induced myocardial hypertrophy in a mouse model of CKD [27]. We found that Klotho suppressed the IS-induced inflammatory response to protect against renal fibrosis and cardiac hypertrophy. In addition, IS reduced the expression of Klotho in the kidneys through activation of NF-ĸB signaling [33]. Overexpression of Klotho downregulated IS-induced phosphorylation of NF-kB p65 *in vitro* and *in vivo*, also suggesting that Klotho overexpression could alleviate heart failure and kidney damage by inactivating the NF-kB pathway.

The inflammatory response plays an important role in the pathogenesis of AKI and CKD [34, 35]. In the early stages of AKI, macrophages infiltrate the kidney and contribute to kidney damage by producing proinflammatory factors [36]. Macrophages are also critical for tissue regeneration and wound healing [36]. M1 macrophages predominate over M2 macrophages during the early stages of AKI and promote inflammation [37]. M1 macrophages produce high levels of pro-inflammatory cytokines including TNFα, IL-6, and IL-1β. In contract, M2 macrophages produce high levels of anti-inflammatory cytokines such as IL-10 to promote tissue regeneration and wound healing [35, 38]. We found that IS induced upregulation of TNFα, IL-6, and IL-1β, and suppressed IL-10 production in macrophages to promote M1 polarization. Our findings indicate overexpression of Klotho in macrophages can restore IL-10 expression and promote macrophage M2 polarization in kidney and heart tissue to alleviate IS-induced heart failure and kidney damage.

Renal fibrosis is always the common final outcome of CKD [39]. Meanwhile, CVD is a serious complication in patients with CKD, which is clinically identified by the high prevalence of left ventricular hypertrophy [40]. Our data indicated that IS obviously induced renal fibrosis and cardiomyocyte hypertrophy in CKD mice model. In contrast, overexpression of Klotho significantly inhibited renal fibrosis and cardiomyocyte hypertrophy induced by IS in mice. Moreover, evidence has been shown that the levels of BUN and serum CR will be significantly increased in patients with CKD [41]. Meanwhile, BNP increase was associated with rapid decline of renal function in patients with CKD and heart failure [42]. In this study, we found that IS markedly increased the levels of serum CR, BUN, and BNP in CKD mice model, which were obviously reversed by Klotho treatment. Meanwhile, overexpression of Klotho markedly promoted macrophage polarization from M1 to M2 in kidney and heart tissues, compared with IS treatment group. These data indicated that overexpression of Klotho alleviated IS-induced heart failure and kidney damage *in vivo* via increasing M2 macrophage polarization.

LPS was previously demonstrated to promote M1 macrophage polarization through activating the NF-κB pathway in THP-1 cells [43]. In addition, curcumin was found to inhibit cisplatin-induced kidney inflammation via inhibiting M1 macrophage polarization and NF-kB activation [44]. We have demonstrated that overexpression of Klotho promotes M2 macrophage polarization to alleviate heart failure and kidney damage in mice by inactivating the NF-kB pathway. Thus, restoring Klotho expression could be an alternative treatment option for CKD patients with heart disease.

## MATERIALS AND METHODS

### Cell culture

THP-1 human acute monocytic leukemia cells were obtained from the American Type Culture Collection (ATCC, Rockville, MD, USA). Cells were incubated in RPMI 1640 medium supplemented with 10% heat-inactivated fetal bovine serum (FBS, Thermo Fisher Scientific, Waltham, MA, USA) and antibiotic-antimycotic solution (100 U/ml penicillin and 0.1 mg/ml streptomycin, Thermo Fisher Scientific) at 37°C in a humidified atmosphere containing 5% CO_2_. Differentiation of THP-1 monocytes into macrophages was induced using PMA (Sigma Aldrich, St. Louis, MO, USA) [45].

### Cell transfection

For *in vitro* studies, the entire human Klotho gene (NM_004795.3) was amplified by PCR using specific oligonucleotide primers that included XhoI and BamHI restriction sites. The PCR product was then digested with XhoI (Takara, Bio, Otsu, Japan) and BamHI (Takara), and then inserted into the pIRES2-ZsGreen1 plasmid (Clontech, Mountain View, CA, USA). The pIRES2-ZsGreen1-Klotho plasmid was then transfected into THP-1-derived macrophages using the Lipofectamine 2000 reagent (Thermo Fisher Scientific) according to the manufacturer’s instructions.

For *in vivo* studies, the Klotho sequence was synthesized by GenePharma (Shanghai), and then sub-cloned into the pDC315-C-FLAG adenovirus expression vector. 293T cells were then transfected with either the NC or pDC315-Klotho-FLAG (Klotho-OE) plasmids. Supernatants were collected after 48 h. THP-1 cells were then seeded into 60-mm cell plates at a density of 2 x 10^5^ cells/mL and incubated overnight. The Klotho-OE supernatants were added to the THP-1 cells and the cells incubated for 24 h. Stable THP-1 cells were then selected by treatment with puromycin (2.5 μg/mL, Sigma Aldrich) for three days.

### Quantitative real-time PCR (RT-qPCR)

Total RNA was extracted from THP-1 cells using the TRIzol reagent (Takara) according to the manufacturer's instructions. The cDNA was synthesized using a cDNA Reverse Transcription Kit (Thermo Fisher Scientific). Real-time PCR was performed with the SYBR Premix Ex Taq II kit (TaKaRa, Dalian, China) using an ABI PRISM® 7500 Sequence Detection System (Applied Biosystems, Foster, CA, USA). The qRT-PCR protocol was the following: 95°C for 5 min followed by 45 cycles of 95°C for 15 s, 60°C for 45 s, and 72°C for 30 s. The following primers were used: Klotho, Forward: 5’- TCACCATCGACAACCCCTAC-3’; Reverse: 5’-GATGCTGTGGTCGGTCATTC-3’. GAPDH, Forward: 5’-CAAGAAGGTGGTGAAGCAGG-3; Reverse: 5’-TCAAAGGTGGAGGAGTGGGT-3’. The relative expression of Klotho was calculated using the 2^-ΔΔCT^ method. GAPDH was used as an internal control.

### Enzyme-linked immunosorbent assays (ELISA)

THP-1 cells were incubated in the presence of PMA (160 nM) for 48 h. After that, the cells were incubated in PMA-free medium for 24 h, and then transfected with the Klotho expression plasmid in the presence or absence of 2 mM IS for 24 h. Cell culture supernatants were then collected and the levels of IL-10, TNFα, IL-6, and IL-1β cytokines quantified by ELISA (ExCellBIO, Shanghai China) according to the manufacturer’s protocol. In addition, the levels of BUN and CR in serum and urine of mice were analyzed using Urea and Creatinine Assay Kits, respectively (Nanjing Jiancheng Bioengineering Institute, Jiangsu, China), according to the manufacturer’s protocols. Furthermore, the level of B-type natriuretic peptide (BNP) in serum of mice was analyzed using the brain natriuretic peptide assay kit according to the manufacturer’s protocol.

### Western blot

Protein concentrations were estimated using the BCA Protein Assay Kit (Thermo Fisher Scientific). Equal quantities of protein lysates (20 μg) were separated by 10% SDS-PAGE and then electro-transferred onto polyvinylidene fluoride membranes (PVDF, Millipore, Billerica, MA, USA). The membranes were blocked with 5% non-fat milk in TBST for 1 h at room temperature, and then incubated overnight at 4°C with the following antibodies: Arginase1 (1: 1000, Abcam Cambridge, MA, USA), iNOS (1: 1000, Abcam), Klotho (1: 1000, Abcam), α-SMA (1: 1000, Abcam), p-p65 (1: 1000, Abcam), p65 (1: 1000, Abcam), GAPDH (1: 1000, Abcam). The following day, the membranes were washed and then incubated with the corresponding secondary antibodies (1:5000, Abcam) for 2 h at room temperature. Proteins were visualized using an ECL Substrate Reagent Kit (Thermo Fisher Scientific).

### Flow cytometry

Flow cytometry was performed using a FACSAria II instrument (BD Biosciences, Franklin Lake, NJ, USA). Data were analyzed using the FACSDiva 6.1.1 software (BD Biosciences). Cells were stained with anti-HLA-DR (M1, macrophage cell subpopulation marker, Abcam) and anti-CD206 (M2, macrophage cell subpopulation marker, Abcam) for 20 min at 4°C. The cells were washed twice with PBS and resuspended in FACS Buffer prior to fluorescence activated cell sorting.

### Animal studies

C57BL/6 mice (10-week-old, n = 24) were purchased from Beijing Vital River Laboratory Animal Technology Co., Ltd (Beijing, China). All animal protocols were approved by the Ethics Committees of Zhejiang Hospital and performed in accordance with the National Institutes of Health Guide for the Care and Use of Animals. Animals were divided into four groups (sham, CKD, CKD + IS, and CKD + IS + Klotho). Mice in the sham group underwent laparotomy and surgical exposure of the kidney. CKD was induced by uninephrectomy as described [17]. IS was administered via intraperitoneal injection at a dose of 100 mg/kg per day [46]. Ad-Klotho 2×10^9^ PFU was administered via tail vein injection on days 1 and 14. The mice were sacrificed under anesthesia after 2 weeks, and kidney and heart tissue collected and weighed.

### Immunofluorescence

Kidney tissue samples were fixed in 4% paraformaldehyde and embedded in paraffin. Specimens were then cut into 4 μm thick sections and incubated with the following antibodies: anti-F4/80 (1: 100, Abcam), anti-Klotho (1: 100, Abcam), and anti-p65 (1: 100, Abcam) overnight at 4°C. Following the incubation, the specimens were stained with Cy3 AffiniPure Goat Anti-Mouse IgG (H+L) (1:250, Amyjet Scientific, Hubei, China) or Goat Anti-Rabbit IgG H&L (FITC) (1:5000; Abcam) for 2 h at room temperature. Cell nuclei were counterstained with DAPI for 15 min. The cells were imaged using a laser scanning confocal microscope (LSM, Carl Zeiss).

### Hematoxylin and eosin staining

Heart tissue samples were fixed in 4% paraformaldehyde and embedded in paraffin. Specimens were then cut into 4 μm thick sections, stained with hematoxylin and eosin, and visualized using a laser scanning confocal microscope (LSM, Carl Zeiss).

### Masson staining

Masson’s Trichrome Stain Kit (Sigma Aldrich) was used to detect collagen and other fibers in kidney tissue as described [47].

### Ultra-performance liquid chromatography (UPLC)

The samples were injected into an Agilent C-18 column (4.6 × 250 mm, 5-μm particle size, Agilent) at 30 °C. The injection volume was 10 μL. The mobile phase flow rate was 1 mL/min. Mobile phase A consisted of 10 mmol ammonium dihydrogen phosphate in water and mobile phase B consisted of hexane nitrile. Gradient elution was performed as follows: 0–10 min, 82% A; 10–20 min, 55% A.

### Statistical analysis

All statistical analysis was performed with GraphPad Prism 7 (GraphPad Software, Inc., La Jolla, CA, USA). Data are reported as the mean ± standard deviation (SD). All experiments were repeated at least three times. Comparisons between multiple groups were performed using one-way analysis of variance (ANOVA) followed by Tukey’s test. A P < 0.05 was considered significant.
