# Correction for: Klotho alleviates indoxyl sulfate-induced heart failure and kidney damage by promoting M2 macrophage polarization

**DOI:** 10.18632/aging.205114

**Published:** 2023-09-29

**Authors:** Jing Lv, Jin Chen, Minjia Wang, Fei Yan

**Affiliations:** 1 Department of General Practice, Zhejiang Hospital, Hangzho, Zhejiang 310013, P.R. China; 2 Department of Critical Care Medicine, Zhejiang Hospital, Hangzho, Zhejiang 310013, P.R. China

**Keywords:** chronic kidney disease, cardiovascular disease, Klotho, indoxyl sulfate, macrophage polarization

**This article has been corrected:** The authors found that in the flow cytometry plots shown in **Figure 3I**, some of the data selected for the M1-HLA-DR and M2-CD206 groups were not representative. To replace those images and confirm their earlier results, they repeated the flow cytometry experiments. The results of those experiments were consistent with the previously acquired data, and the authors selected new representative images for Figure 3I. Corrected **Figure 3** is presented below.

**Figure 3:**
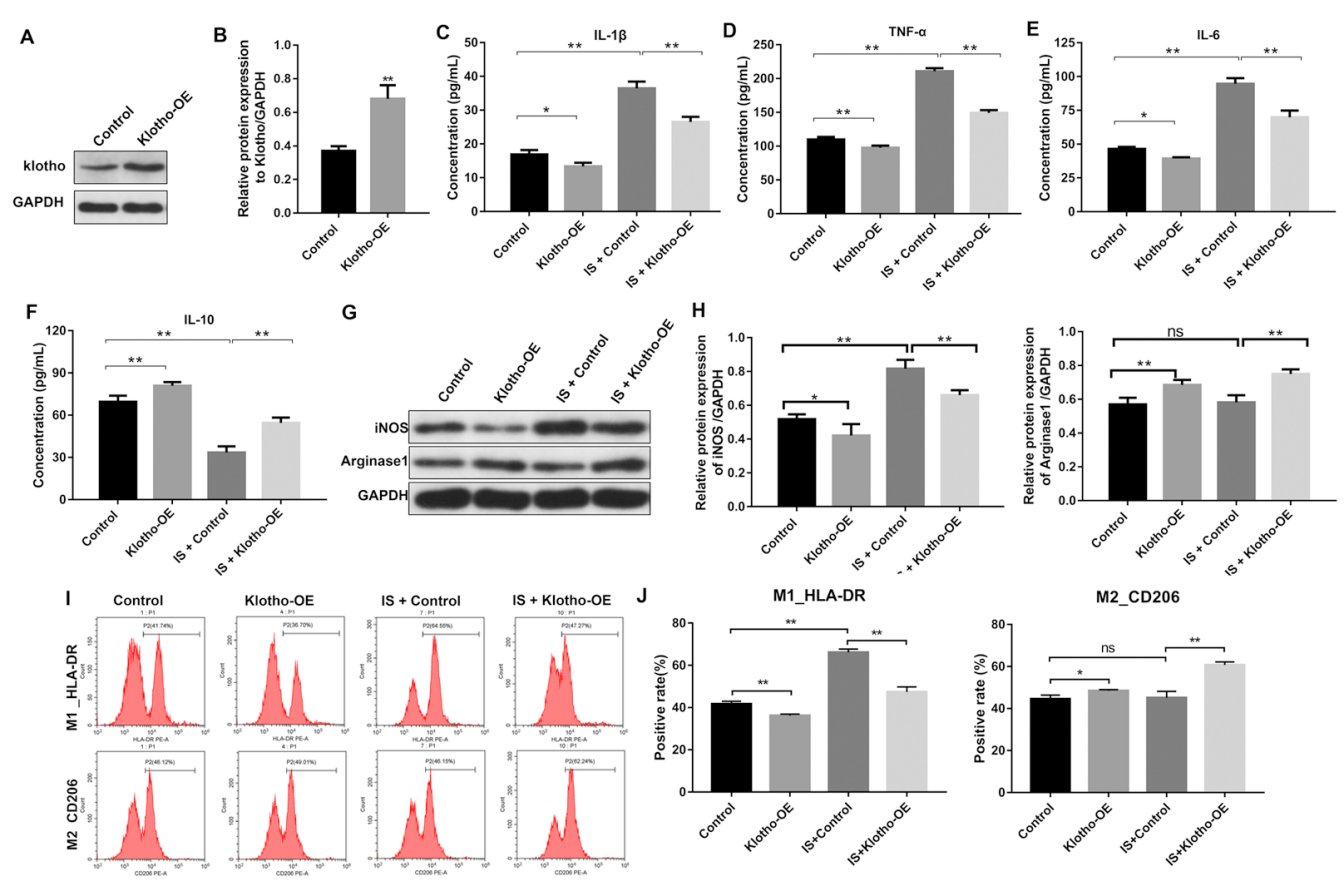
**Overexpression of Klotho suppresses the IS-induced inflammatory response in macrophages by stimulating M2 polarization. **(**A**) THP-1 cells were exposed to PMA (160 nM) for 48 h, incubated in PMA-free medium for 24 h, and then transfected with the Klotho expression plasmid for 24 h. Klotho expression in cells was evaluated by western blotting. (**B**) Klotho expression was normalized to GAPDH. (**C**–**F**) THP-1 cells were exposed to PMA (160 nM) for 48 h, incubated in PMA-free medium for 24 h. Subsequently, cells were transfected with Klotho expression plasmid or treated with 2 mM IS for 24 h respectively. Meanwhile, cells were transfected with Klotho expression plasmid for 24 h in the presence of 2 mM IS. The levels of IL-10, IL-6, TNFα, and IL-1β in cells were evaluated by ELISA. (**G**) The expression of iNOS and Arginase1 in cells was analyzed by western blotting. (**H**) The expression of iNOS and Arginase1 in cells was normalized to GAPDH. (**I**) Representative FACS plots for HLA-DR, a marker of M1 macrophages, and CD206, a marker of M2 macrophages. (**J**) The percentages of HLA-DR- and CD206+ cells were detected by FACS. *P < 0.05 and **P < 0.01.

